# Case Report: Root resorption caused after pulp death of adjacent primary molar

**DOI:** 10.12688/f1000research.15375.1

**Published:** 2018-08-03

**Authors:** Maha M. Azab, Dalia M. Moheb, Osama I. El Shahawy

**Affiliations:** 1 Department of Pediatric Dentistry, Faculty of Dentistry, Fayoum University, Fayoum, Egypt; 2 Department of Pediatric Dentistry, Faculty of Dentistry, Cairo University, Cairo, Egypt

**Keywords:** Root resorption, Necrotic tooth, Pulpectomy

## Abstract

Necrotic decayed primary molars with necrotic pulp tissues may show periapical involvement and root resorption. In this case report, a pediatric patient with a very common chief complain and clinical picture of necrotic badly decayed molar, introduced a very interesting case when radiographic investigation was performed, which showed that root resorption of the adjacent healthy molar occurred. The current report is, to the best of our knowledge, the first to report such finding in primary dentition.

## Introduction

Root resorption is the physiologic or pathologic loss of dentin and/or cementum and/or bone ^1^.

Primary teeth can go through either type, but other than resorption during the shedding process resorption is considered pathologic. Inflammatory root resorption is not a rare finding in the pediatric community, with spread of infection from a carious tooth as a main cause ^2, 3^. In the present case, the interesting finding is that root resorption did not only occur in the carious, necrotic tooth but also occurred in the adjacent vital tooth.

## Case report

A seven and half year-old boy visited the outpatient clinic of Pediatric Dentistry Department, Faculty of Dentistry, Cairo University in June 2015 with a chief complaint of pain on the lower right molar area. The patient’s mother stated that the pain was at times throbbing in nature, and child is not able to chew on this side.

Clinical examination showed a badly decayed, lower second primary molar with related localized intraoral abscess, where the lower first primary molar was intact. The patient had poor oral hygiene; he had not received any professional dental care, and was very apprehensive.

**Figure 1.**
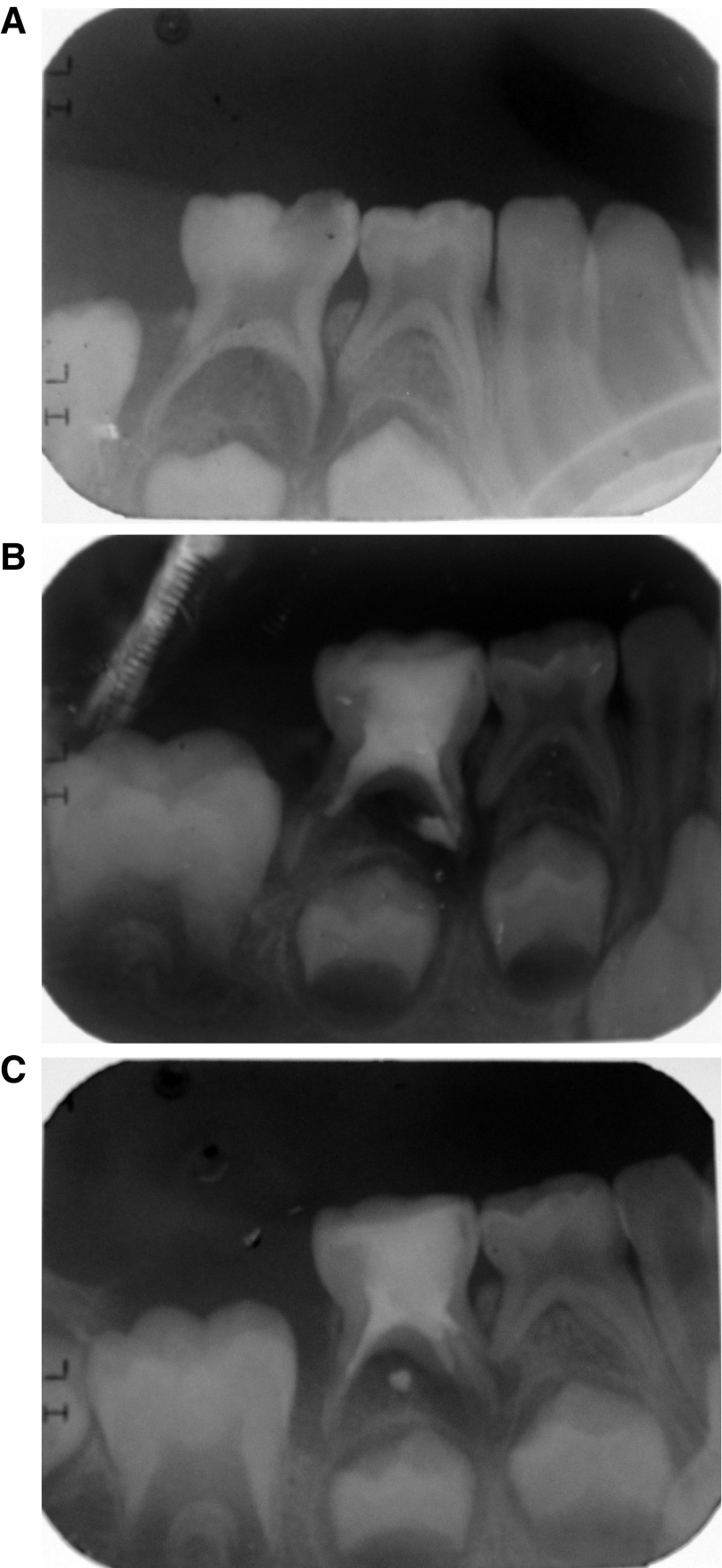
Radiographic examination. **A**) Pre-operative radiograph: Bone rarefaction and root resorption in first and second primary molars; **B**) Post-operative radiograph: Pulpectomy treatment in lower second primary molar; **C**) 8 months post-operative: cessation of root resorption.

Radiographic examination revealed root resorption and bone rarefaction related to lower second primary molar. The interesting finding was a considerable amount of root resorption of the distal root of the adjacent lower first primary molar ( Figure 1A).

The case was managed by performing pulpectomy ^4^ to the lower second primary molar, with root canals filled with calcium hydroxide paste with iodoform (Metapex, Meta Biomed, Republic of Korea). The tooth was then restored with high viscosity glass ionomer (GC Fuji IX GP capsule, GC corporation, Tokyo, Japan) ( Figure 1B). The lower first primary molar was not touched and instead monitored. No antibiotics or analgesics was prescribed.

Unfortunately, the patient’s mother did not want follow-up appointments in person, however, she was contacted on the phone, after 2 weeks, 3 months and 6 months, and she said everything was fine and there was no swelling or pain.

At about 8 months from the treatment appointment, the patient’s mother visited the outpatient clinic with the patient for other reasons, and decided to pass by the Pediatric Dentistry Department for patient follow-up. Clinical examination showed no signs or symptoms, occlusal restoration was intact, and radiographic examination revealed arrested root resorption, on both molars, and an increase in the density of bone although this was not at a normal level yet ( Figure 1C).

Table 1 shows the patient’s timeline of symptoms, treatment and follow-up.

**Table 1.** Case timeline.

Time	Event	
**0**	Patient visited clinic, history, clinical and radiographic examination	Abscess related to lower right second primary molar, and root resorption in distal root of adjacent first primary molar
**0**	Pulpectomy procedure	
**+ 2 weeks**	1 ^st^ Follow up (Phone inquiry)	No pain, no swelling (symptom free)
**+ 3 months**	2 ^nd^ follow up (Phone inquiry)	No pain, no swelling (symptom free)
**+ 6 months**	3 ^rd^ follow up (Phone inquiry)	No pain, no swelling (symptom free)
**+ 8 months**	4 ^th^ follow up (clinical and radiographic assessment)	No pain, no swelling (symptom free). Arrested root resorption, Improvement of bone density.

## Discussion

Caries-related inflammatory root resorption is caused when bacteria from infected pulp stimulate resorptive cells, thus removal of infected pulp is necessary for cessation of the condition ^5^.

The only previously reported similar case was a periapical lesion adjacent to a tooth with failing root canal therapy, where healing did not occur till extraction of the adjacent tooth ^6^. 

In the current case, the treatment choice for the lower second primary molar was obvious and clear. The problem with the adjacent tooth, which was intact but suffered from root resorption, is that it is not clear by signs, symptoms and investigation whether the root resorption is just caused (due to proximity) by resorptive cells stimulated from bacteria from the necrotic pulp chamber of lower second primary molar, or if bacteria or bacterial toxins have spread to the lower first primary molar, causing retrograde infection, which would have necessitated pulp therapy to the first primary molar as well.

We have chosen the more conservative treatment plan, which involved the pulpectomy of lower second primary molar and follow-up for the lower first primary molar, which turned out to be appropriate, where mother reported.

## Patient perspective

The patient’s mother was pleased with the more conservative treatment performed, as the child was very apprehensive, and she preferred the least clinical procedure possible. She and the child were satisfied with the results as clinical symptoms subsided after treatment.

## Conclusion

Although a rare finding, one should consider the possibility of root resorption caused by periapical infection of adjacent tooth, when no other symptoms are present, as the least invasive treatment and follow-up should be tried first.

## Consent

Written informed consent for publication of the clinical details and images was obtained from the patient's mother.

## Data availability

All data underlying the results are available as part of the article and no additional source data are required.

## References

[1] Ne RF Witherspoon DE Gutmann JL: Tooth resorption. *Quintessence Int.* 1999;30(1):9–25. 10323155

[2] Santos BZ Bosco VL Silva JYB: Physiological and pathological factors and mechanisms in the process of root resorption of deciduous teeth. *RSBO (Online).* 2010;7(3):332–9. Reference Source

[3] Vieira-Andrade RG Drumond CL Alves LP: Inflammatory root resorption in primary molars: prevalence and associated factors. *Braz Oral Res.* 2012;26(4):335–40. 10.1590/S1806-83242012000400009 22790498

[4] Bharuka SB Mandroli PS: Single- versus two-visit pulpectomy treatment in primary teeth with apical periodontitis: A double-blind, parallel group, randomized controlled trial. *J Indian Soc Pedod Prev Dent.* 2016;34(4):383–90. 10.4103/0970-4388.191429 27681404

[5] Finucane D Kinirons MJ: External inflammatory and replacement resorption of luxated, and avulsed replanted permanent incisors: a review and case presentation. *Dent Traumatol.* 2003;19(3):170–4. 10.1034/j.1600-9657.2003.00154.x 12752540

[6] Frank AL: Inflammatory resorption caused by an adjacent necrotic tooth. *J Endod.* 1990;16(7):339–41. 10.1016/S0099-2399(06)81946-1 2081949

