# Supplementary material for: Case Report: Root resorption caused after pulp death of adjacent primary molar
Source: F1000Res. 2018 Aug 3;7:1186. [Version 1] doi: 10.12688/f1000research.15375.1 (PMC6124378; doi:10.12688/f1000research.15375.1)
Supplement: Peer review history

# Peer review: Referee response for version 1

**DOI:** 10.5256/f1000research.16754.r37166

**Published:** 2018-08-31

**Authors:** Zafer C. Cehreli

**Affiliations:** 1 Department of Pediatric Dentistry, Faculty of Dentistry, Hacettepe University, Ankara, Turkey

This paper reports a very rare occurrence of the spreading of periapical infection to a healthy primary molar root. The non-vital tooth causing the infection was treated endodontically without any intervention to the vital neighboring primary molar. After 8 months, arrest of the resorption was evident along with improved periapical healing. 

 Minor comments:

1. Please discuss the effect of coronal restoration on the prognosis of endodontic treatment.
2. Please discuss the fate of extruded root canal medicament.

I have read this submission. I believe that I have an appropriate level of expertise to confirm that it is of an acceptable scientific standard.

# Peer review: Referee response for version 1

**DOI:** 10.5256/f1000research.16754.r36785

**Published:** 2018-08-08

**Authors:** Mariem O. Wassel

**Affiliations:** 1 Department of Pediatric Dentistry and Dental Public Health, Faculty of Dentistry, Ain Shams University, Cairo, Egypt

1. The title can be changed to "primary molar root resorption after pulp death of an adjacent primary molar" to clarify that root resorption occurred to an adjacent primary tooth not a permanent one.
2. Start the abstract with decayed primary molars, delete "necrotic"
3. Indicate in abstract the age of the child and that resorption occurred in an adjacent primary molar 
4. Replace root resorption by inflammatory root resorption in key words
5. Give hint about child's medical history 
6. Write the details of pulpectomy (single or 2 visits), irrigation used
7. Why is SSC not fitted?
8. In ref 6 cited text (page 3, first paragraph), please specify  whether this was a primary or permanent tooth
9. In conclusion, please start a new sentence from "when no other symptoms are present........." and delete "as" in the same sentence

I have read this submission. I believe that I have an appropriate level of expertise to confirm that it is of an acceptable scientific standard.
